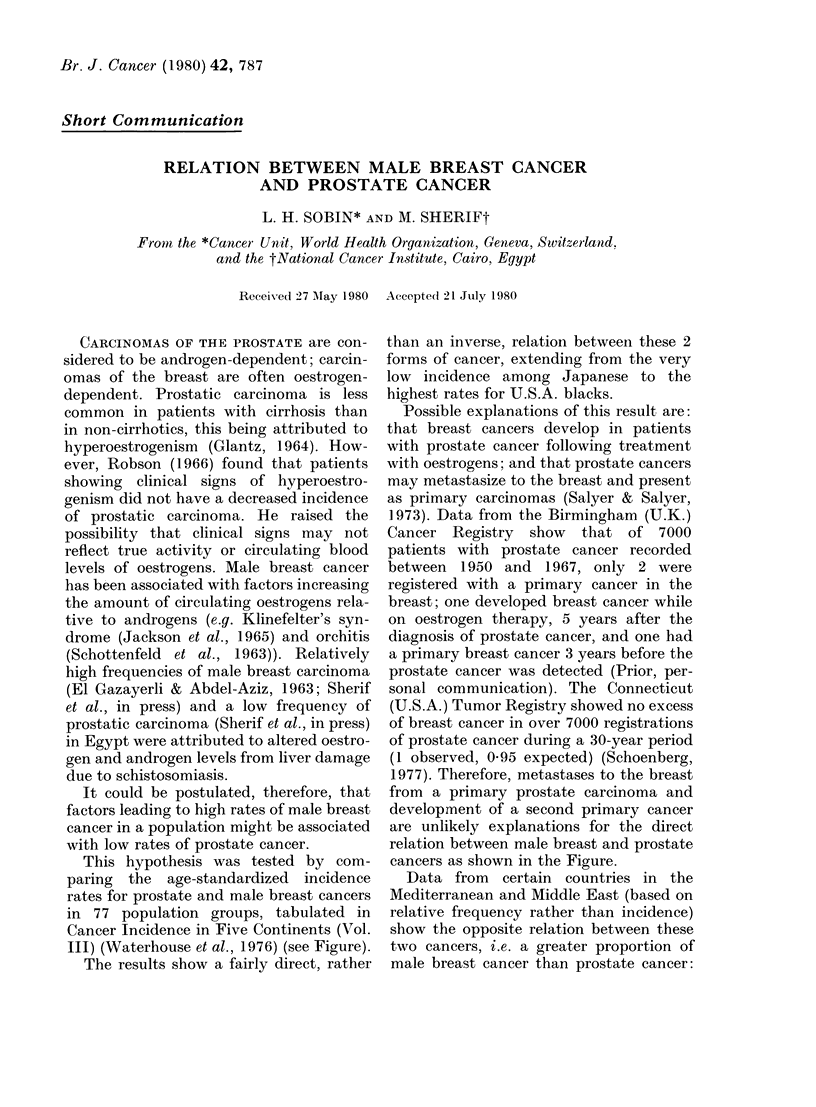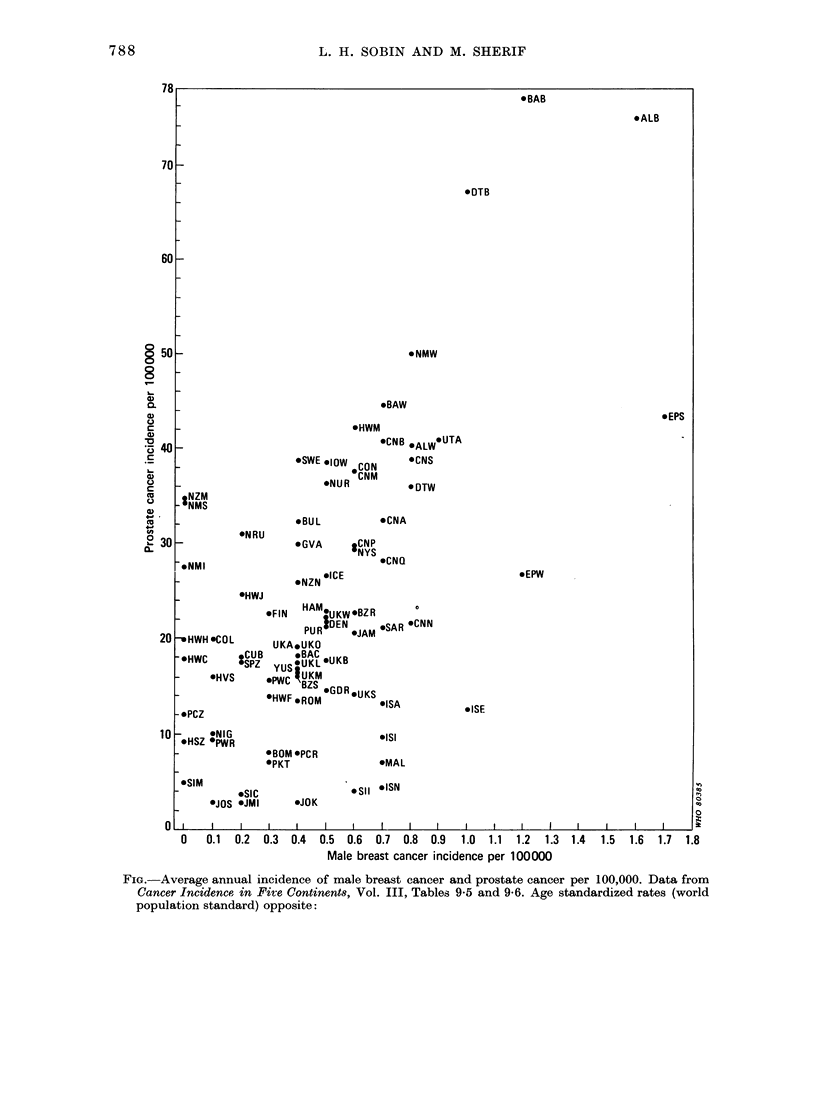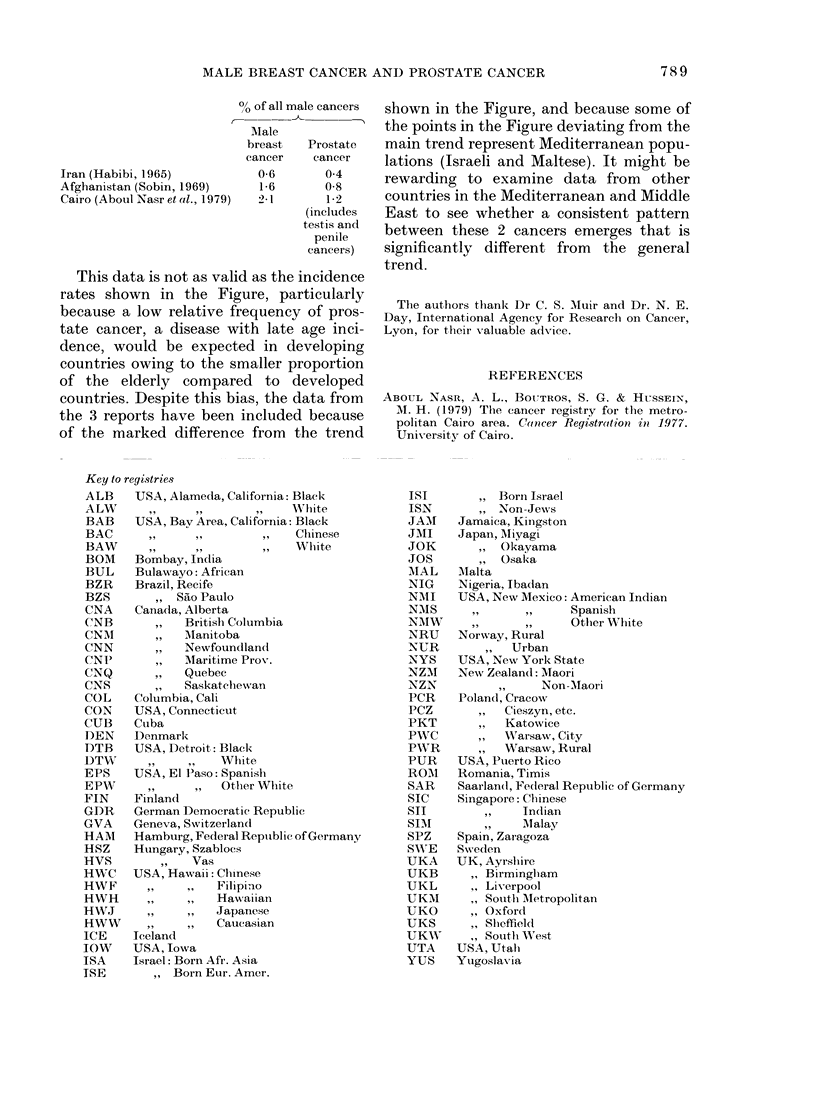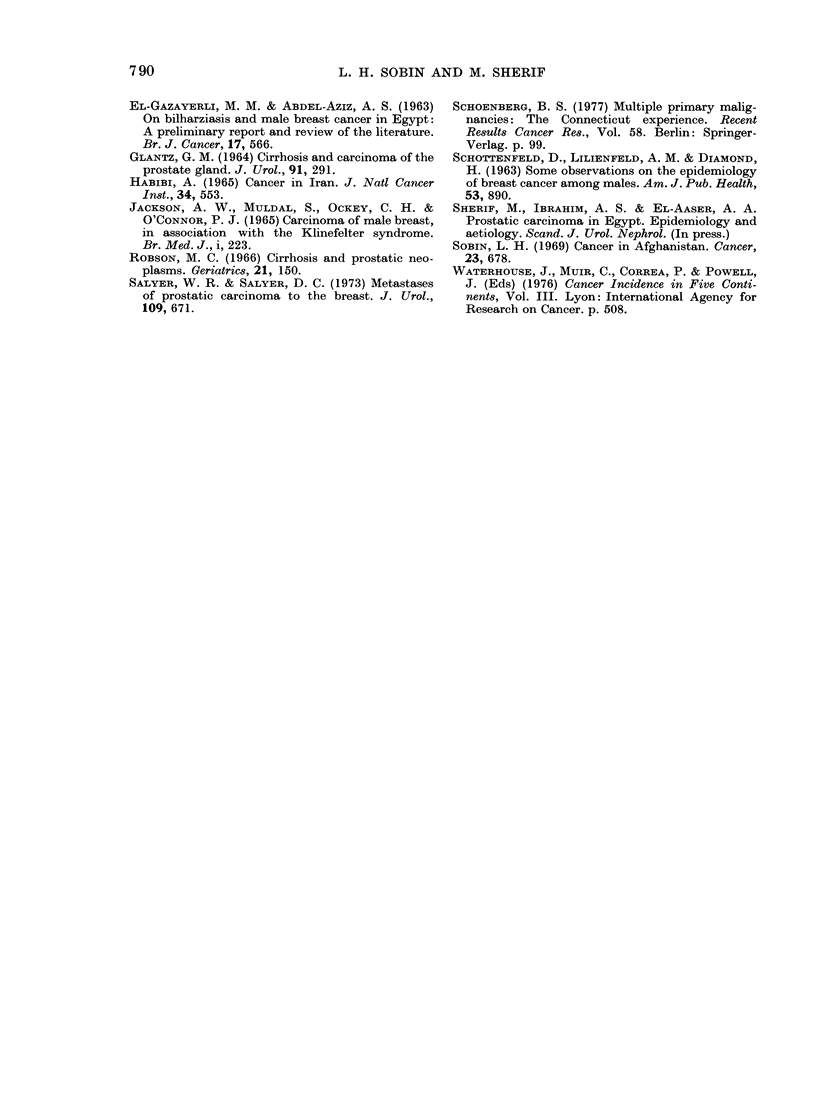# Relation between male breast cancer and prostate cancer.

**DOI:** 10.1038/bjc.1980.313

**Published:** 1980-11

**Authors:** L. H. Sobin, M. Sherif


Br. J. Cancer (1980) 42, 787

### Short Communication

### RELATION BETWEEN MALE BREAST CANCER AND PROSTATE CANCER

L. H. SOBIN* AND M. SHERIF†

From the *Cancer Unit, World Health Organization, Geneva, Switzerland, and the †National Cancer Institute, Cairo, Egypt

Received 27 May 1980 Accepted 21 July 1980

CARCINOMAS OF THE PROSTATE are considered to be androgen-dependent; carcinomas of the breast are often oestrogen-dependent. Prostatic carcinoma is less common in patients with cirrhosis than in non-cirrhotics, this being attributed to hyperoestrogenism (Glantz, 1964). However, Robson (1966) found that patients showing clinical signs of hyperoestrogenism did not have a decreased incidence of prostatic carcinoma. He raised the possibility that clinical signs may not reflect true activity or circulating blood levels of oestrogens. Male breast cancer has been associated with factors increasing the amount of circulating oestrogens relative to androgens (*e.g.* Klinefelter's syndrome (Jackson *et al.*, 1965) and orchitis (Schottenfeld *et al.*, 1963)). Relatively high frequencies of male breast carcinoma (El Gazayerli & Abdel-Aziz, 1963; Sherif *et al.*, in press) and a low frequency of prostatic carcinoma (Sherif *et al.*, in press) in Egypt were attributed to altered oestrogen and androgen levels from liver damage due to schistosomiasis.

It could be postulated, therefore, that factors leading to high rates of male breast cancer in a population might be associated with low rates of prostate cancer.

This hypothesis was tested by comparing the age-standardized incidence rates for prostate and male breast cancers in 77 population groups, tabulated in Cancer Incidence in Five Continents (Vol. III) (Waterhouse *et al.*, 1976) (see Figure).

The results show a fairly direct, rather than an inverse, relation between these 2 forms of cancer, extending from the very low incidence among Japanese to the highest rates for U.S.A. blacks.

Possible explanations of this result are: that breast cancers develop in patients with prostate cancer following treatment with oestrogens; and that prostate cancers may metastasize to the breast and present as primary carcinomas (Salyer & Salyer, 1973). Data from the Birmingham (U.K.) Cancer Registry show that of 7000 patients with prostate cancer recorded between 1950 and 1967, only 2 were registered with a primary cancer in the breast; one developed breast cancer while on oestrogen therapy, 5 years after the diagnosis of prostate cancer, and one had a primary breast cancer 3 years before the prostate cancer was detected (Prior, personal communication). The Connecticut (U.S.A.) Tumor Registry showed no excess of breast cancer in over 7000 registrations of prostate cancer during a 30-year period (1 observed, 0·95 expected) (Schoenberg, 1977). Therefore, metastases to the breast from a primary prostate carcinoma and development of a second primary cancer are unlikely explanations for the direct relation between male breast and prostate cancers as shown in the Figure.

Data from certain countries in the Mediterranean and Middle East (based on relative frequency rather than incidence) show the opposite relation between these two cancers, *i.e.* a greater proportion of male breast cancer than prostate cancer:

FIG.—Average annual incidence of male breast cancer and prostate cancer per 100,000. Data from *Cancer Incidence in Five Continents*, Vol. III, Tables 9·5 and 9·6. Age standardized rates (world population standard) opposite:

| | % of all male cancers | |
|---|---|---|
| | Male breast cancer | Prostate cancer |
| Iran (Habibi, 1965) | 0·6 | 0·4 |
| Afghanistan (Sobin, 1969) | 1·6 | 0·8 |
| Cairo (Aboul Nasr *et al.*, 1979) | 2·1 | 1·2 (includes testis and penile cancers) |

This data is not as valid as the incidence rates shown in the Figure, particularly because a low relative frequency of prostate cancer, a disease with late age incidence, would be expected in developing countries owing to the smaller proportion of the elderly compared to developed countries. Despite this bias, the data from the 3 reports have been included because of the marked difference from the trend shown in the Figure, and because some of the points in the Figure deviating from the main trend represent Mediterranean populations (Israeli and Maltese). It might be rewarding to examine data from other countries in the Mediterranean and Middle East to see whether a consistent pattern between these 2 cancers emerges that is significantly different from the general trend.

The authors thank Dr C. S. Muir and Dr. N. E. Day, International Agency for Research on Cancer, Lyon, for their valuable advice.

REFERENCES

Aboul Nasr, A. L., Boutros, S. G. & Hussein, M. H. (1979) The cancer registry for the metropolitan Cairo area. *Cancer Registration in 1977.* University of Cairo.

*Key to registries*

| | |
|---|---|
| ALB | USA, Alameda, California: Black |
| ALW | ,, ,, ,, White |
| BAB | USA, Bay Area, California: Black |
| BAC | ,, ,, ,, Chinese |
| BAW | ,, ,, ,, White |
| BOM | Bombay, India |
| BUL | Bulawayo: African |
| BZR | Brazil, Recife |
| BZS | ,, São Paulo |
| CNA | Canada, Alberta |
| CNB | ,, British Columbia |
| CNM | ,, Manitoba |
| CNN | ,, Newfoundland |
| CNP | ,, Maritime Prov. |
| CNQ | ,, Quebec |
| CNS | ,, Saskatchewan |
| COL | Columbia, Cali |
| CON | USA, Connecticut |
| CUB | Cuba |
| DEN | Denmark |
| DTB | USA, Detroit: Black |
| DTW | ,, ,, White |
| EPS | USA, El Paso: Spanish |
| EPW | ,, ,, Other White |
| FIN | Finland |
| GDR | German Democratic Republic |
| GVA | Geneva, Switzerland |
| HAM | Hamburg, Federal Republic of Germany |
| HSZ | Hungary, Szablocs |
| HVS | ,, Vas |
| HWC | USA, Hawaii: Chinese |
| HWF | ,, ,, Filipino |
| HWH | ,, ,, Hawaiian |
| HWJ | ,, ,, Japanese |
| HWW | ,, ,, Caucasian |
| ICE | Iceland |
| IOW | USA, Iowa |
| ISA | Israel: Born Afr. Asia |
| ISE | ,, Born Eur. Amer. |
| ISI | ,, Born Israel |
| ISN | ,, Non-Jews |
| JAM | Jamaica, Kingston |
| JMI | Japan, Miyagi |
| JOK | ,, Okayama |
| JOS | ,, Osaka |
| MAL | Malta |
| NIG | Nigeria, Ibadan |
| NMI | USA, New Mexico: American Indian |
| NMS | ,, ,, Spanish |
| NMW | ,, ,, Other White |
| NRU | Norway, Rural |
| NUR | ,, Urban |
| NYS | USA, New York State |
| NZM | New Zealand: Maori |
| NZN | ,, Non-Maori |
| PCR | Poland, Cracow |
| PCZ | ,, Cieszyn, etc. |
| PKT | ,, Katowice |
| PWC | ,, Warsaw, City |
| PWR | ,, Warsaw, Rural |
| PUR | USA, Puerto Rico |
| ROM | Romania, Timis |
| SAR | Saarland, Federal Republic of Germany |
| SIC | Singapore: Chinese |
| SII | ,, Indian |
| SIM | ,, Malay |
| SPZ | Spain, Zaragoza |
| SWE | Sweden |
| UKA | UK, Ayrshire |
| UKB | ,, Birmingham |
| UKL | ,, Liverpool |
| UKM | ,, South Metropolitan |
| UKO | ,, Oxford |
| UKS | ,, Sheffield |
| UKW | ,, South West |
| UTA | USA, Utah |
| YUS | Yugoslavia |

El-Gazayerli, M. M. & Abdel-Aziz, A. S. (1963) On bilharziasis and male breast cancer in Egypt: A preliminary report and review of the literature. *Br. J. Cancer*, **17**, 566.

Glantz, G. M. (1964) Cirrhosis and carcinoma of the prostate gland. *J. Urol.*, **91**, 291.

Habibi, A. (1965) Cancer in Iran. *J. Natl Cancer Inst.*, **34**, 553.

Jackson, A. W., Muldal, S., Ockey, C. H. & O'Connor, P. J. (1965) Carcinoma of male breast, in association with the Klinefelter syndrome. *Br. Med. J.*, i, 223.

Robson, M. C. (1966) Cirrhosis and prostatic neoplasms. *Geriatrics*, **21**, 150.

Salyer, W. R. & Salyer, D. C. (1973) Metastases of prostatic carcinoma to the breast. *J. Urol.*, **109**, 671.

Schoenberg, B. S. (1977) Multiple primary malignancies: The Connecticut experience. *Recent Results Cancer Res.*, Vol. 58. Berlin: Springer-Verlag. p. 99.

Schottenfeld, D., Lilienfeld, A. M. & Diamond, H. (1963) Some observations on the epidemiology of breast cancer among males. *Am. J. Pub. Health*, **53**, 890.

Sherif, M., Ibrahim, A. S. & El-Aaser, A. A. Prostatic carcinoma in Egypt. Epidemiology and aetiology. *Scand. J. Urol. Nephrol.* (In press.)

Sobin, L. H. (1969) Cancer in Afghanistan. *Cancer*, **23**, 678.

Waterhouse, J., Muir, C., Correa, P. & Powell, J. (Eds) (1976) *Cancer Incidence in Five Continents*, Vol. III. Lyon: International Agency for Research on Cancer. p. 508.